# Renal protection CT protocol using low-dose and low-concentration iodine contrast medium in at-risk patients of HCC and with chronic kidney disease: a randomized controlled non-inferiority trial

**DOI:** 10.1186/s40644-023-00616-0

**Published:** 2023-10-19

**Authors:** Jeong Hee Yoon, Jin Young Park, Sang Min Lee, Eun Sun Lee, Jae Hyun Kim, Jeong Min Lee

**Affiliations:** 1 Department of Radiology, Seoul National University Hospital and College of Medicine, 101 Daehak-ro, Jongno-gu, Seoul, 03080 Korea; 2 Department of Radiology, Seoul National University College of Medicine, 103 Daehak-ro, Jongno-gu, Seoul, 03087 Republic of Korea; 3 Department of Radiology, Inje University Busan Paik Hospital, Bokji-ro 75, Busangjin-gu, Busan, 47392 Republic of Korea; 4 Department of Radiology, CHA Gangnam Medical Center, CHA University, 566 Nonhyun-ro, Gangnam-gu, Seoul, 06135 Republic of Korea; 5 Department of Radiology, Chung-Ang University Hospital, Seoul, 06973 Republic of Korea; 6 Institute of Radiation Medicine, Seoul National University Medical Research Center, 103 Daehak-ro, Jongno-gu, Seoul, 03087 Republic of Korea

## Abstract

**Background:**

Although efforts have been made to reduce the dose of Contrast Medium (CM) to improve patient safety, there are ongoing concerns regarding its potential effects on image quality and diagnostic performance. Moreover, research is lacking to establish a lower limit for safe and effective CM dose reduction. To determine whether the image quality of contrast-enhanced liver computed tomography (CT) using a reduced amount of iodinated CM was similar to that of standard liver CT.

**Methods:**

We enrolled participants at risk for hepatocellular carcinoma with decreased estimated glomerular filtration rates (< 60 mL/min/1.73m^2^). Participants were randomly assigned to the standard group or the renal protection protocol (RPP) group. In the standard group, images were reconstructed using hybrid iterative reconstruction (iDose), while in the RPP group, low monoenergetic (50-keV) images and deep learning (DL)-based iodine-boosting reconstruction were used. Four radiologists independently assessed image quality and lesion conspicuity.

**Results:**

Fifty-two participants were assigned to the standard (n = 25) or RPP (n = 27) groups. The iodine load was significantly lower in the RPP group than in the standard group (301.5 ± 1.71 vs. 524 ± 7.37 mgI/kg, *P* < 0.001). The 50-keV and DL-based iodine-boosting images from the RPP group exhibited higher image contrast than those from the standard group during arterial (3.60 ± 0.65, 3.75 ± 0.60, and 3.09 ± 0.43, respectively) and portal venous phases (4.01 ± 0.49, 3.86 ± 0.42, and 3.21 ± 0.31, respectively) (*P* < 0.05 for all). Overall image quality was superior in the RPP group (*P* < 0.05 for all). No significant difference in lesion conspicuity was observed (*P* > 0.017).

**Conclusions:**

The reduction in image contrast and overall image quality caused by decreased CM can be restored using either low monoenergetic imaging or DL-based iodine-boosting reconstruction.

**Trial registration:**

clinicaltrials.gov, NCT04024514, Registered July 18, 2019, prospectively registered, https://classic.clinicaltrials.gov/ct2/show/NCT04024514.

**Supplementary Information:**

The online version contains supplementary material available at 10.1186/s40644-023-00616-0.

## Introduction

Hepatocellular carcinoma (HCC) constitutes approximately 80% of primary liver tumors [1]. Intrahepatic recurrence following curative surgical or non-surgical treatment can occur in ≥ 70% of patients within 5 years [2], necessitating regular follow-up even after treatment. The imaging modalities employed during follow-up include ultrasound, contrast-enhanced computed tomography (CECT), and magnetic resonance imaging (MRI). Of these, CECT is the most commonly used due to its advantages in the identification of recurrence risk, cost, examination time, and the detection of extrahepatic metastases. To achieve an adequate contrast-to-noise ratio (CNR) in the arterial phase, the use of high concentrations of iodinated contrast medium (CM) is often required [3]. Although recent studies have argued that the risk of contrast-induced acute kidney injury (AKI) has been overstated due to confounding factors, substantial risk undeniably exists for patients with severely compromised renal function [4], patients with diminished renal function who are under surveillance for HCC recurrence, iodinated CM administration is often avoided because the development of AKI in patients with cirrhosis is not uncommon and frequently indicates a poor prognosis [5, 6]. While ultrasound is often utilized as a primary surveillance modality in patients at risk of developing HCC [7, 8], it is not typically recommended in patients with prior HCC due to a higher risk of developing HCC compared with those without prior HCC [9, 10]. Additionally, contrast-enhanced MRI is often not preferred due to concerns regarding the potential for irreversible nephrogenic systemic fibrosis [11].

Several studies have reported the use of enhanced CNR with reduced amounts of CM, achieved by techniques such as low peak kilovoltage (kVp) or low virtual monoenergetic images of dual-energy CT approaches [12–15]. However, much of that research focuses on CT angiography, where soft tissue contrast is of less importance, whereas relatively few reports have explored the feasibility of abdominal CT. Notably, while low virtual monoenergetic images can enhance iodine contrast, they are also impacted by an increase in noise. This issue can be mitigated using specific noise reduction strategies, such as the spatial frequency separation algorithm or anti-correlated noise reduction [16–18]. Nevertheless, virtual monoenergetic images can only be generated through dual-energy CT, which may be unfeasible for hospitals that rely on conventional single-energy CT scanners. Recently, a deep learning (DL)-based iodine-boosting method using a 120-kVp single-energy CT scanning mode has shown promise in reducing the CM dose without diminishing the detection rate of HCC compared with standard CM dose acquisition [19].

Considering the possible advantages of the low monoenergetic technique and the DL-based iodine-boosting algorithm in reducing the CM dose, our study focused on patients with chronic kidney disease. Our objective was to determine whether the image quality in liver CECT, conducted with a decreased amount of iodinated CM, was non-inferior in patients with impaired renal function compared to the standard liver CT protocol.

## Methods

This prospective single-center randomized study was approved by the Institutional Review Board of Seoul National University Hospital, and written informed consent was obtained from all participants (NCT04024514). Financial support was provided by Riyeon Pharmaceuticals (Seoul, South Korea); however, the authors retained full control over the data and information submitted for publication.

### Participants

Between December 2019 and December 2020, we enrolled participants who met the following eligibility criteria: (a) older than 20 years; (b) at high risk of developing HCC (chronic hepatitis B, C, or liver cirrhosis except for congestive hepatopathy) and scheduled for liver CT for diagnostic or follow-up purposes; (c) decreased renal function (estimated glomerular filtration rate [eGFR] < 60 mL/min/1.73 m^2^) and not on dialysis; and (d) provided informed consent. Exclusion criteria were as follows: (a) younger than 20 years; (b) not at high risk for developing HCC; (c) on dialysis; (d) no venous access in the forearm; (e) anticipated beam hardening artifacts due to a prosthesis; and (f) any relative or absolute contraindication of CECT except renal dysfunction. The eGFR was calculated according to the CKD-EPI 2009 formula [20]: eGFR = 141 × min (serum creatinine [Cr]/κ, 1)^α^ × max (serum Cr/κ, 1)^−1.209^ × 0.993^age^ × 1.018 [if female] × 1.159 [if Black], where κ is 0.7 for female and 0.9 for male patients, and α is − 0.329 for female and − 0.411 for male patients.

### Participant assignments

Participants were randomly assigned to either the standard liver CT protocol group (the standard group) or the renal protection protocol liver CT group (the RPP group). A computer-generated permuted block randomization process, managed by our medical research collaboration center, was utilized. The block sizes were 4 and 6 for a 1:1 allocation. Two stratification factors were employed by research coordinators: (a) BMI (< 25 kg/m^2^ vs. ≥ 25 kg/m^2^) and (b) eGFR (< 45 mL/min/1.73 m^2^ vs. ≥ 45 mL/min/1.73 m^2^). Participants, investigators, and outcome assessors were all blinded to the participant allocation.

### CT examination

All participants were asked to fast for at least 6 h prior to the CECT examination. Intravenous hydration was administered before CT scan, and oral hydration was encouraged after examination in accordance with our institutional protocol to prevent CM-induced nephrotoxicity. All CT scans were conducted using a dual-layer CT scanner (IQon; Philips Healthcare, Amsterdam, The Netherlands) with the following settings: 120 kVp, a gantry rotation time of 0.33 s, 64 × 0.625 mm collimation, and a slice thickness of 3 mm with 2-mm reconstruction intervals. The precontrast, portal venous, and delayed phases were acquired before, 70 s after, and 180 s after CM administration, respectively. The arterial phase was obtained using the bolus tracking technique, initiated 17 s after reaching a trigger threshold of 150 Hounsfield units at the abdominal aorta. The scan parameters remained consistent across all four phases.

In our study, we used 525 mgI/kg of iodinated CM for the standard liver CT protocol, while 300 mgI/kg was used for the RPP liver CT. In the standard group, iodinated CM (Ioversol 350 mgI/mL, Optiray350; Riyeon Pharmaceuticals) was administered via a power injector (Stellant®; Medrad, Pittsburgh, PA, USA) for 35 s, followed by a 30-mL saline flush. In the RPP group, iodinated CM with a lower iodine concentration (Ioversol 320 mgI/mL, Optiray320; Riyeon Pharmaceuticals) was used in the same manner, with the minimum contrast injection rate set at 2 mL/sec. In both groups, the maximum volume of administered CM was limited to one vial (130 mL) according to our institutional safety protocol.

### Image reconstruction

In the standard group, CT images were reconstructed using a hybrid iterative reconstruction (iDose level 4). In the RPP group, arterial, portal, and delayed phase images were reconstructed using a monoenergetic image (50 keV) and a DL-based iodine-boosting method, in addition to the automatically generated iDose images of precontrast, arterial, portal, and delayed phases.

*DL-based iodine enhancement* — DL-based reconstruction was carried out using commercially available software (ClariACE; ClariPi, Seoul, South Korea). This method employs a two-stage U-net architecture, in which image denoising and contrast augmentation occur sequentially. Initially, the DL-based denoising algorithm extracts a noise component image from the input image (iDose in this study), which is then subtracted from the input image [21, 22]. Next, the augmentation process begins, during which the iodine component image is extracted using the DL-based iodine enhancement algorithm. Ultimately, the extracted iodine component image is added to the denoised image, thereby improving the signal-to-noise ratio (SNR) and contrast-to-noise ratio (CNR) of the augmented CECT image [19].

### Image analysis

*Qualitative analysis —* Four fellowship-trained body radiologists (J. H. Y., E. S. L., J. Y. P., and S. M. L., with 12, 12, 8, and 8 years of experience after fellowship, respectively) independently reviewed the images, ensuring that the window width and level were always adjustable. Image noise, image contrast, image texture, and overall image quality were scored on a 5-point scale for both arterial and portal venous phases, with higher scores indicating better image quality (Table E1). The location and size of non-cystic focal liver lesions (FLLs), excluding lipiodol uptake, were also documented. Lesion conspicuity during the arterial and portal venous phases was scored on a 5-point scale as follows: 1, not visible (automatically assigned to missed lesions); 2, barely delineated; 3, visible with a blurry margin; 4, visible lesion with a relatively sharp margin and acceptable contrast; and 5, clear contrast and a sharp margin [13].

*Quantitative analysis* — One fellowship-trained body radiologist (J.H.Y.) drew three circular regions of interest (ROIs) on consecutive slices at the level of the celiac trunk in the aorta during the arterial phase and at the main portal vein during the portal venous phase. Additionally, three ROIs were drawn in the liver parenchyma at the hilar level, avoiding vessels and FLLs. The average Hounsfield unit value was used as a representative value for the aorta, main portal vein, and liver parenchyma. Finally, circular ROIs were drawn in the subcutaneous fat layer of the anterior abdominal wall on three consecutive slices during both the arterial and portal venous phases. The standard deviation of the ROI values was considered to represent the image noise for each phase.

### Reference standard

Two fellowship-trained radiologists (J. M. L. and J. H. K., with 20 and 4 years of experience, respectively, after fellowship) who did not participate in the review session reviewed the images to establish the reference standard for FLL detection. This reference standard was based on the most recent follow-up imaging, including a standard dose of liver CT or liver MRI taken within 3 months for patients with LR-3, -4, -5, or -M observations. For both groups, interval cancers identified during follow-up were considered true FLLs that had been missed on CECT. For those with no FLLs or only benign FLLs, remote cross-sectional images were used instead of 3-month follow-up images. A comprehensive description of the reference standard can be found in the supplementary material 1. In brief, HCC was diagnosed using histology or typical imaging characteristics on CECT, CE-MRI, CE-ultrasound, or tumor staining on angiography for transarterial chemoembolization. Benign FLLs were confirmed based on the stability of size on follow-up imaging and the imaging features.

### Study outcome

The primary endpoint is to prove non-inferiority of image quality in RPP group compared with standard group. Secondary outcome is to compare the lesion conspicuity and detection rates between the two groups. Lastly, we collected information of participant-reported anuria, follow-up creatinine level and eGFR within a month after CT when available.

### Statistical analysis

The sample size was determined based on a retrospective study examining improved image quality in monoenergetic images with reduced CM (4.3 ± 0.6 in monoenergetic images vs. 3.6 ± 0.3 in standard images) [14]. We assumed a one-sided significance level of 0.05, a target power of 0.95, and an allocation ratio of 1 for each group, along with a non-inferiority margin of − 0.2, as informed by a previous study of low-dose CT angiography [23]. As a result, the minimum number of participants required for each group was 22, and the final sample size was set at 52, considering an 18% dropout rate.

The independent-samples *t*-test was conducted to assess the differences between the two groups. To evaluate differences based on reconstruction methods within the same group, we employed either the paired *t*-test or the Friedman test, as appropriate. The intraclass correlation coefficient (ICC) was calculated to determine the inter-observer agreement. Due to the presence of multiple lesions in a patient, lesion conspicuity was analyzed using the generalized estimating equation method. A normal distribution and the identity link function were applied, and lesion conspicuity was reported with a 95% confidence interval (CI).

To compare the detection rate of FLLs between groups, we estimated the reader-averaged figure of merit (FOM) using the random reader, random lesion method in a weighted free response receiver operating characteristic analysis. This considered multiple lesions and lesion locations within the same participant. FLLs were considered to have been detected by each reviewer if the lesion conspicuity score was 2 or higher. Lesion conspicuity and detection were evaluated for all participants and in subgroups stratified by FLL size (less than 20 mm vs. 20 mm or greater).

All statistical analyses were conducted using commercially available software packages (SAS version 9.4, SAS Institute Inc., Cary, NC, USA; SPSS version 27, IBM Corp., Armonk, NY, USA; MedCalc version 20.216, MedCalc Software, Ostend, Belgium) and R version 4.1.1 (R Foundation for Statistical Computing, Vienna, Austria). A p-value of less than 0.05 was considered to indicate a statistically significant difference.

## Results

**Fig. 1:**
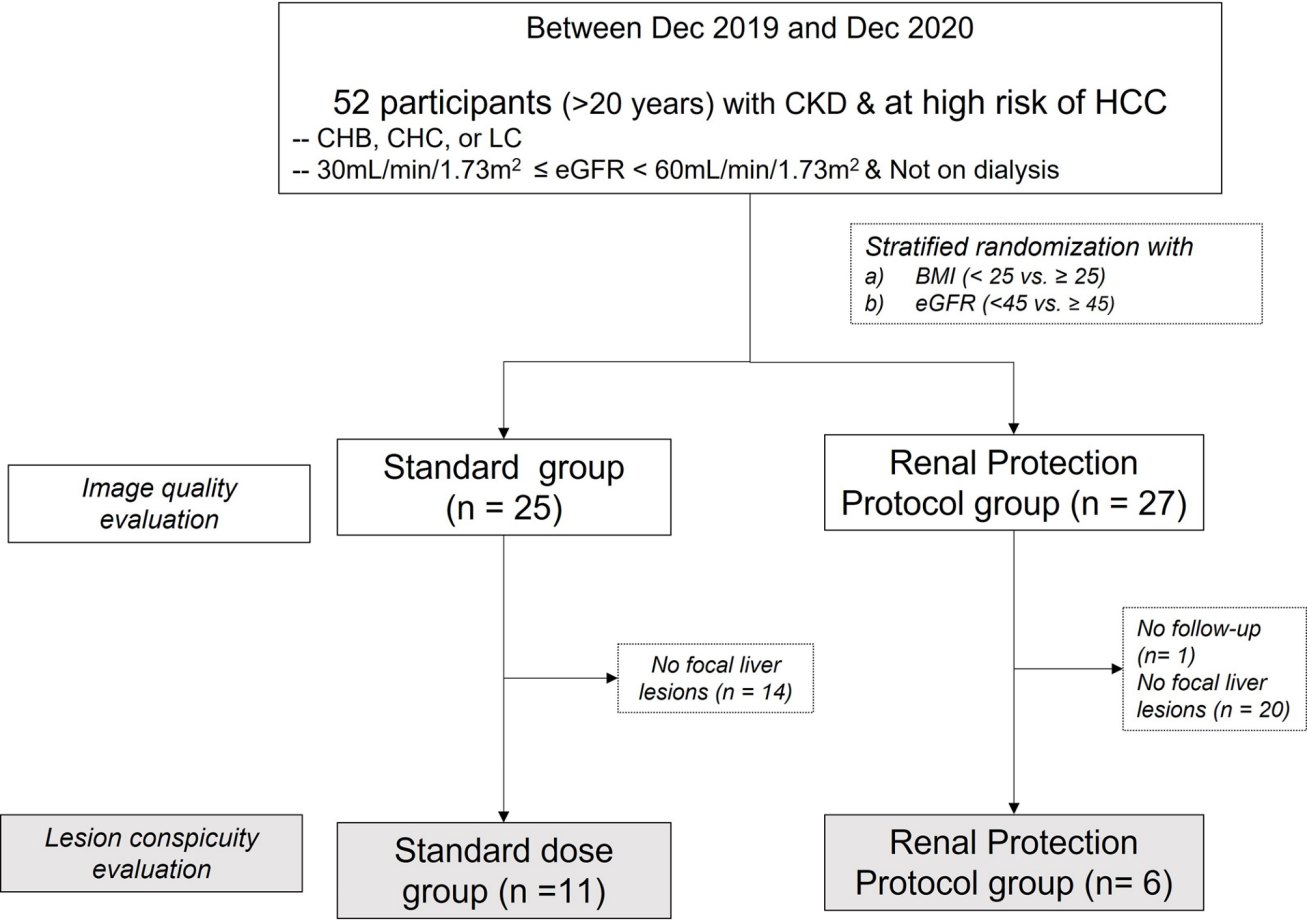
Study flow. BMI = body mass index, CHB = chronic hepatitis **B,** CHC = chronic hepatitis **C,** CKD = chronic kidney disease, eGFR = estimated glomerular filtration rate, HCC = hepatocellular carcinoma, LC = liver cirrhosis

A total of 52 participants (43 male, mean age 71.9 ± 9.2 years) were enrolled and randomly assigned to the standard group (n = 25; 19 male, mean age 73 ± 8.6 years) and the RPP group (n = 27; 24 male, mean age 71 ± 9.9 years) (Fig. 1). 23% (12/52) of participants had eGFR < 45 mL/min/1.73 m^2^. No statistically significant differences were observed between the two groups in terms of age, weight, body mass index, baseline eGFR, and serum creatinine levels (*P* > 0.05 for all, Table 1). A total of 62 FLLs were identified in both groups (47 in the standard group and 15 in the RPP group). In the standard group, 44% (11/25) of participants had 47 non-cystic FLLs: 61.7% (29/47) were HCCs, followed by 31.9% with dysplastic nodules (15/47), 2.1% with hemangiomas (1/47), 2.1% with post-inflammatory granulomas (1/47), and 2.1% with vascular malformations (1/47). In the RPP group, 22.2% (6/27) of participants presented with 15 non-cystic FLLs; 73.3% (11/15) were HCCs and 26.7% (4/15) were dysplastic nodules. The average FLL size was 15.1 ± 14.8 mm (range, 5–89 mm) in the standard group and 25.9 ± 20.0 mm (9–76 mm) in the RPP group (p = 0.028). FLLs smaller than 20 mm constituted 89.3% (42/47) of the FLLs in the standard group and 60% (9/15) of those in the RPP group.

**Table 1:** Demographic characteristics

	Standard group	Renal protection protocol group	*P*-value
**Sex** Male: female	19:6	24:3	0.224
**Age (years)**	73 ± 8.6	71 ± 9.9	0.438
**Weight (kg)**	64.7 ± 9.8 (51, 93)	68.6 ± 10.2 (52, 97)	0.178
**Body mass index (kg/m** ^**2**^ **)**	25.3 ± 4.1 (19.2, 35.4)	25.3 ± 3.1 (18.7, 34)	0.942
**Underlying disease**			0.076
CHB CHC Alcoholic LC Cryptogenic LC NASH-LC	80 (20) 8 (2) 8 (2) 0 (0) 4 (1)	51.9 (14) 11.1 (3) 18.5 (5) 18.5 (5) 0 (0)	0.076
**Comorbidities**			
Diabetes mellitus Hypertension Coronary disease Stroke Non-HCC malignancy	40 (10) 32 (8) 8 (2) 4 (1) 36 (9)	44.4 (12) 22.2 (6) 3.7 (1) 3.7 (1) 11.1 (3)	0.918 0.432 0.511 0.956 0.035
**Medications**			
ACE inhibitor or ARB Beta-blocker Diuretic Immunosuppressant NSAID	48 (12) 32 (8) 24 (6) 4 (1) 20 (5)	40.7 (11) 22.2 (6) 18.5 (5) 7.4 (2) 14.8 (4)	0.602 0.432 0.632 0.602 0.625
**Child-Pugh class**			
CLD or class A Class B or C	76 (19) 24 (6)	81.5 (22) 18.5 (5)	0.881
**eGFR (mL/min/1.73 m** ^**2**^ **)**	50.14 ± 6.6 (34.6, 59.4)	49.12 ± 7.14 (30.2, 59.2)	0.593
**Serum creatinine**	1.34 ± 0.24 (0.94, 1.98)	1.37 ± 0.33 (0.70, 2.40)	0.790
**CM amount (mL)** ^**†**^	96.8 ± 13.7 (77, 130)	64.6 ± 9.5 (49, 91)	< 0.001
**Iodine load (mg/kg)** ^**†**^	524.0 ± 7.37 (489.3*, 528.4)	301.5 ± 1.71 (298, 306.6)	< 0.001
**DLP (mGy∙cm)**	893.9 ± 197.1 (690.4, 1455.1)	929.7 ± 217.2 (704.7, 1782.7)	0.538

Note: Values are given as number (percentage) or mean ± standard deviation (range). ACE = angiotensin-converting enzyme, ARB = angiotensin receptor blocker, CHB = chronic hepatitis B, CHC = chronic hepatitis C, CLD = chronic liver disease, CM = contrast medium, DLP = dose-length product, eGFR = estimated glomerular filtration rate, HCC = hepatocellular carcinoma, LC = liver cirrhosis, NASH = non-alcoholic steatohepatitis, NSAID = non-steroidal anti-inflammatory drug

*P*-value < 0.05 indicates a statistically significant difference between the two groups

*In one participant, the estimated CM amount (140 mL) exceeded 1 vial of CM (130 mL), and only one vial was administered per study protocol

^†^We administered CM with different iodine concentrations: 350 mgI/mL for the standard group and 320 mgI/mL for the RPP group

### CM dose in the standard and RPP groups

The administered CM dose was significantly lower in the RPP group than in the standard group (64.6 ± 9.5 mL at 320 mgI/mL vs. 95.6 ± 15.5 mL at 350 mgI/mL; *P* < 0.001) (Table 1). The iodine load was also significantly lower in the RPP group than in the standard group (301.5 ± 1.71 mgI/kg vs. 524 ± 7.37 mgI/kg; *P* < 0.001). In one participant from the standard group, the calculated CM dose (140 mL) exceeded the dose of 1 vial (130 mL), and a 1-vial dose was administered per the study protocol. No significant difference in the dose-length product was observed between the standard and RPP groups (893.9 ± 197.1 vs. 929.7 ± 217.2 mGy·cm, respectively; *P* = 0.538).

### Image quality in the standard and RPP groups

**Fig. 2:**
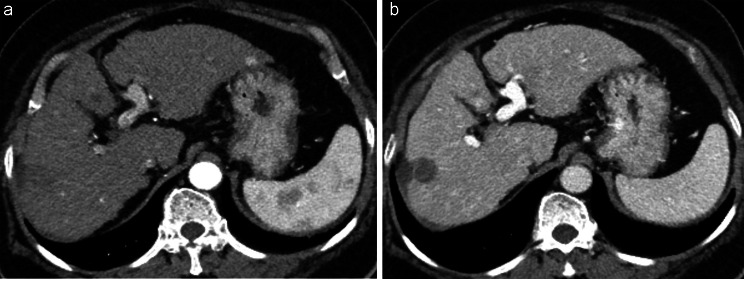
A 70-year-old woman with liver cirrhosis and diabetes in the standard group. The participant’s weight was 70 kg, and her body mass index was 29.4 kg/m^2^. The amount of iodine administered was 525 mg/kg. Arterial **(a)** and portal venous **(b)** phases demonstrated suitable image contrast and acceptable overall image quality

**Fig. 3:**
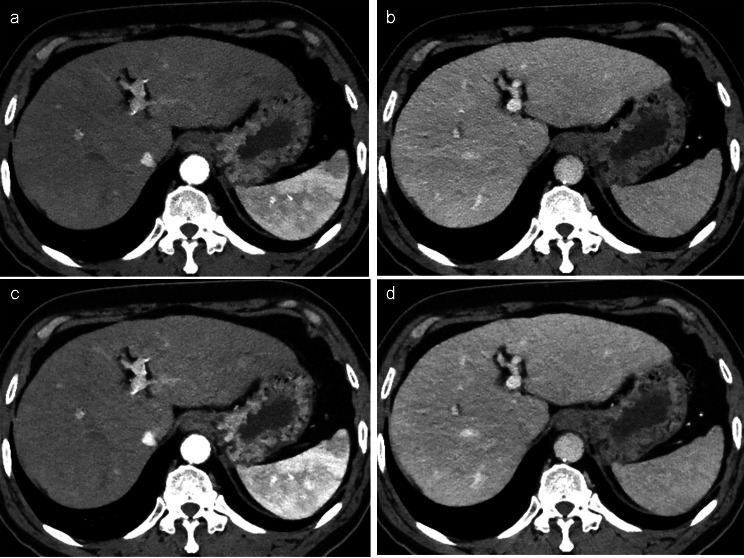
A 63-year-old man with history of HCC and alcoholic liver disease in the RPP group. The participant’s weight was 87 kg, and his body mass index was 29.4 kg/m^2^. The amount of iodine administered was 297.9 mg/kg. Despite the low iodine load, both the arterial **(a)** and portal venous phase **(b)** of the 50-keV images, as well as the arterial **(c)** and portal venous phase **(d)** of the DL-based iodine-boosting reconstructed images, demonstrated sufficient image contrast and acceptable image quality. HCC = hepatocellular carcinoma, RPP = renal protection protocol, DL = deep learning

Compared to the standard group, the 50-keV reconstruction images of the RPP group exhibited higher image contrast in both the arterial (3.09 ± 0.43 vs. 3.60 ± 0.65, respectively; p = 0.002) and portal venous phases (4.01 ± 0.49 vs. 3.21 ± 0.31, respectively; *P* < 0.001). Additionally, the 50-keV images from the RPP group demonstrated less qualitative image noise and higher overall image quality scores in both arterial and portal venous phases than the iDose images from standard CT (*P* < 0.001 for all). Conversely, the 50-keV reconstruction in the RPP group displayed a less satisfactory image texture than the standard group in both arterial (4.06 ± 0.32 vs. 4.48 ± 0.35, respectively) and portal venous phases (4.01 ± 0.49 vs. 4.47 ± 0.31, respectively) (*P* < 0.001 for all) (Figs. 2, 3).

For DL-iodine boosted images, the RPP group demonstrated significantly higher image contrast, lower image noise, and better overall image quality than the standard group (*P* < 0.001 for all, Table 2). However, the DL-based iodine-boosted images exhibited a more artificial texture than the standard group in both the arterial phase (3.56 ± 0.53 vs. 4.48 ± 0.35, respectively) and the portal venous phase (3.50 ± 0.42 vs. 4.47 ± 0.31, respectively; *P* < 0.001 for both).

**Table 2:** Comparison of the qualitative image analysis between the standard and RPP groups

	Standard group (n = 25)	RPP group (n = 27)		*P*-value	
	iDose reconstruction (A)	50-keV reconstruction (B)	DL iodine-boosting reconstruction (C)	(A) vs. (B)	(A) vs. (C)
**Arterial phase**					
Qualitative image noise	3.51 ± 0.30 (3.00, 4.00)	3.93 ± 0.45 (2.50, 4.75)	3.89 ± 0.42 (2.50, 4.75)	< 0.001	0.001
Quantitative image noise	10.4 ± 1.71 (6.13, 13.04)	8.66 ± 1.28 (6.20, 11.42)	7.74 ± 0.95 (6.26, 10.01)	< 0.001	< 0.001
Image contrast	3.09 ± 0.43 (2.25, 4.00)	3.60 ± 0.65 (1.75, 4.50)	3.75 ± 0.60 (1.75, 4.50)	0.002	< 0.001
SNR of aorta	33.63 ± 8.45 (20.66, 59.60)	57.09 ± 14.29 (24.81, 78.55)	67.45 ± 17.66 (26.34, 100.75)	< 0.001	< 0.001
Image texture	4.48 ± 0.35 (3.75, 5.00)	4.06 ± 0.32 (3.50, 4.50)	3.56 ± 0.53 (3.50, 4.50)	< 0.001	< 0.001
Overall image quality	3.20 ± 0.47 (2.25, 4.00)	3.88 ± 0.58 (2.25, 4.75)	3.57 ± 0.56 (2.25, 4.50)	< 0.001	0.012
**Portal venous phase**					
Qualitative image noise	3.60 ± 0.26 (3.25, 4.25)	4.12 ± 0.29 (3.50, 4.75)	3.98 ± 0.27 (3.25, 4.50)	< 0.001	< 0.001
Quantitative image noise	11.22 ± 1.56 (7.99, 14.85)	9.13 ± 1.76 (6.65, 13.40)	8.04 ± 1.05 (6.61, 11.04)	< 0.001	< 0.001
Image contrast	3.21 ± 0.31 (2.75, 4.00)	4.01 ± 0.49 (2.25, 4.75)	3.86 ± 0.42 (3.25, 4.75)	< 0.001	< 0.001
SNR of portal vein	15.90 ± 3.16 (9.99, 24.0)	24.71 ± 5.70 (15.11, 36.54)	29.29 ± 6.30 (19.15, 43.83)	< 0.001	< 0.001
SNR of liver	10.23 ± 2.06 (6.50, 15.42)	14.61 ± 3.39 (6.60, 21.33)	14.61 ± 3.39 (6.60, 21.33)	< 0.001	< 0.001
CNR of liver	5.66 ± 1.75 (2.56, 9.31)	10.11 ± 3.01 (5.56, 15.21)	12.34 ± 3.19 (5.29, 19.45)	< 0.001	< 0.001
Image texture	4.47 ± 0.31 (3.75, 5.00)	4.09 ± 0.23 (3.75, 4.50)	3.50 ± 0.42 (3.00, 4.50)	< 0.001	< 0.001
Overall image quality	3.44 ± 0.36 (2.75, 4.25)	4.16 ± 0.35 (3.25, 4.75)	3.63 ± 0.44 (3.00, 4.50)	< 0.001	0.095

Note: Values are given as mean ± standard deviation (range). p < 0.05 indicates a statistically significant difference. CNR = contrast-to-noise ratio, DL = deep learning, RPP = renal protection protocol, SNR = signal-to-noise ratio. *P*-value < 0.017 indicated a significant difference

In the arterial phase, the ICC was 0.574 (95% CI: 0.453–0.673) for image noise, 0.841 (95% CI: 0.80–0.878) for image contrast, 0.569 (95% CI: 0.447–0.670) for image texture, and 0.814 (95% CI: 0.762–0.858) for overall image quality. In the portal venous phase, the ICC was 0.560 (95% CI: 0.435–0.663) for image noise, 0.911 (95% CI: 0.887–0.932) for image contrast, 0.592 (95% CI: 0.476–0.687) for image texture, and 0.840 (95% CI: 0.795–0.878) for overall image quality.

### Lesion conspicuity in the standard and RPP groups

**Fig. 4:**
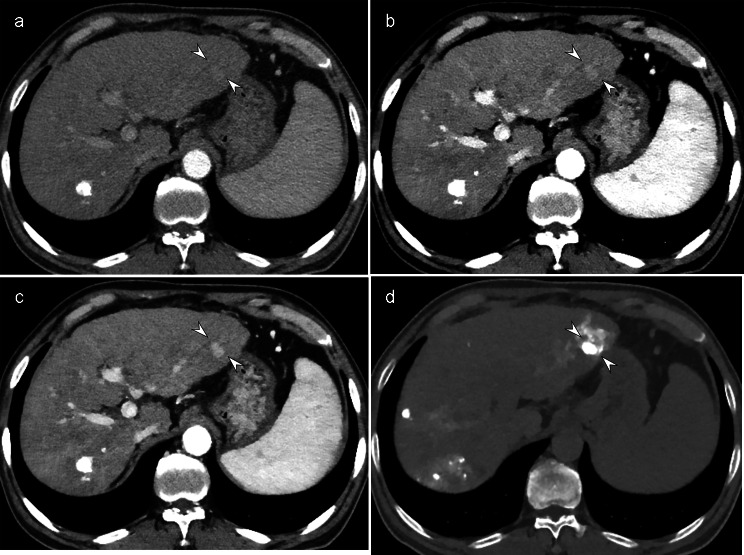
A 71-year-old man with liver cirrhosis in the RPP group. Compared to the iDose image **(a)**, both the 50-keV **(b)** and DL-based iodine-boosting reconstruction **(c)** demonstrated improved lesion conspicuity of a 12-mm HCC in segment 2 (arrowheads), which was subsequently confirmed through angiography and lipiodol uptake **(d**) RPP = renal protection protocol, DL = deep learning, HCC = hepatocellular carcinoma

The standard group exhibited lesion conspicuities of 2.21 in the arterial phase and 2.19 in the portal venous phase (Table 3). The RPP group displayed lesion conspicuities of 2.49 in the arterial phase and 2.56 in the portal venous phase for the 50-keV images, as well as 2.05 in the arterial phase and 2.32 in the portal venous phase for the DL-based iodine-boosting images (Fig. 4). No significant differences in lesion conspicuity were observed between the standard and RPP groups using either 50-keV reconstruction or DL-based iodine-boosting (*P* > 0.05 for all). Additionally, no significant difference in lesion conspicuity was found between groups for small FLLs (< 20 mm).

**Table 3:** Comparison of lesion conspicuity between the standard and RPP groups

	Estimate (95% CI)				Difference (95% CI)			*P*-value		
	Standard group iDose (A)		RPP group 50 keV (B)	RPP group DL-iodine boosting (C)	(A) vs. (B)		(A) vs. (C)	(A) vs.		
								(B)	(C)	
**Arterial phase**										
All FLLs (n = 62)	2.21 (1.24, 3.18)	2.49 (2.14, 2.84)		2.05 (1.80, 2.31)	0.28 (− 0.75, 1.31)	−0.16 (− 1.16, 0.84)		0.596		0.757
< 20 mm (n = 50)	2.13 (1.15, 3.10)	2.15 (2.02, 2.28)		1.85 (1.66, 2.04)	0.02 (− 0.97, 1.00)	−0.28 (− 1.27, 0.72)		0.971		0.586
≥ 20 mm (n = 12)	2.75 (1.88, 3.62)	3.32 (2.79, 3.85)		2.54 (1.97, 3.10)	0.57 (− 0.44, 1.59)	−0.21 (− 1.25, 0.82)		0.269		0.685
**Portal venous phase**										
All FLLs (n = 62)	2.19 (1.34, 3.04)	2.56 (2.04, 3.08)		2.32 (1.87, 2.77)	0.37 (− 0.62, 1.37)		0.13 (− 0.83, 1.09)	0.464		0.787
< 20 mm (n = 50)	2.13 (1.29, 2.96)	2.19 (2.09, 2.29)		1.91 (1.71, 2.12)	0.06 (− 0.78, 0.90)		−0.22 (− 1.08, 0.64)	0.884		0.621
≥ 20 mm (n = 12)	2.60 (1.68, 3.52)	3.46 (2.12, 4.81)		3.32 (2.35, 4.29)	0.86 (− 0.76, 2.49)		0.72 (− 0.61, 2.06)	0.298		0.290

Note: CI = confidence interval, DL = deep learning, FLLs = focal liver lesions, RPP = renal protection protocol. *P*-value < 0.017 indicated a significant difference

### Lesion detection in the standard and RPP groups

In the arterial phase, we observed a significant difference in lesion detection rates between the standard group and the RPP group with 50-keV reconstruction (FOM: 0.594 vs. 0.825, respectively; p = 0.006) (Table 4). The difference in lesion detection rates between the standard group and the RPP group with 50-keV imaging was marginally significant for the portal venous phase (FOM: 0.574 vs. 0.735, *P* = 0.033). No significant differences were found between the standard group and the RPP group with DL-iodine-boosting reconstruction (FOM: 0.742 in the arterial phase, p = 0.163; 0.632 in the portal venous phase, p = 0.216). For small FLLs (< 20 mm), no significant difference was found between the two groups, regardless of the reconstruction method used (*P* = 0.059–0.624, Table 4).

**Table 4:** Comparison of lesion detection rates between the standard and RPP groups

	Figure of merit (95% CI)			*P*-value	
	Standard group iDose (A)	RPP group 50 keV (B)	RPP group DL-iodine boosting (C)	(A) vs. (B)	(A) vs. (C)
**Arterial phase**					
All FLLs (n = 62)	0.594 (0.468, 0.721)	0.825 (0.713, 0.936)	0.742 (0.574, 0.911)	0.006	0.163
< 20 mm (n = 50)	0.574 (0.438, 0.711)	0.735 (0.569, 0.901)	0.632 (0.506, 0.758)	0.059	0.524
**Portal venous phase**					
All FLLs (n = 62)	0.606 (0.476, 0.735)	0.800 (0.675, 0.925)	0.747 (0.563, 0.931)	0.033	0.216
< 20 mm (n = 50)	0.577 (0.452, 0.702)	0.698 (0.596, 0.800)	0.620 (0.491, 0.749)	0.086	0.624

Note: CI = confidence interval, FLLs = focal liver lesions, RPP = renal protection protocol. Figure of merit was not calculated for FLLs ≥ 20 mm due to the small number of FLLs (n = 12). *P*-value < 0.017 indicated a significant difference

### Comparison of reconstruction algorithms in the RPP group

**Fig. 5:**
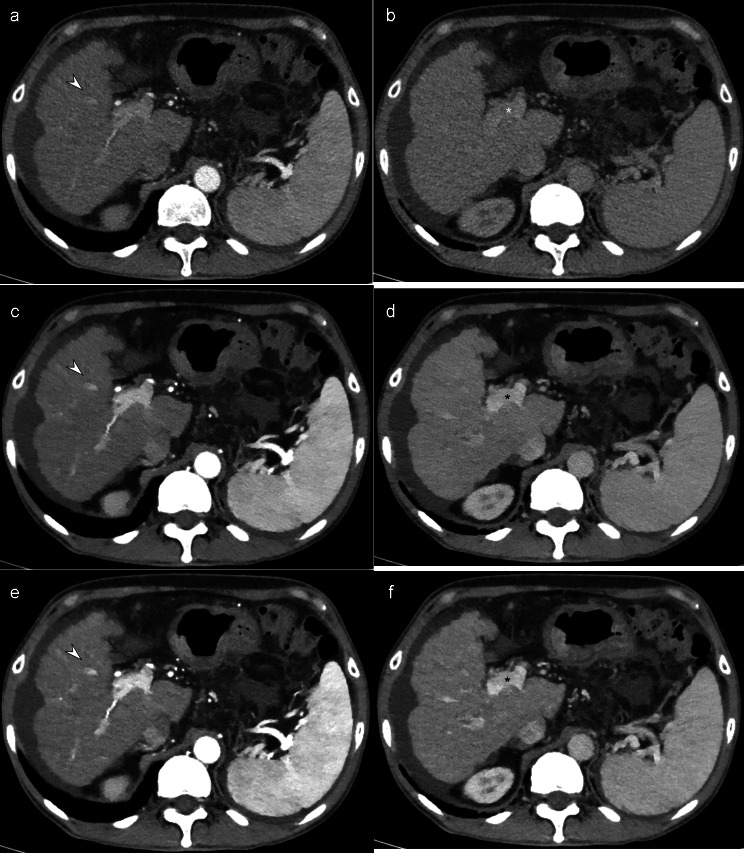
A 68-year-old man with liver cirrhosis in the RPP group. The amount of iodine administered was 301.5 mg/kg. Compared to the iDose image (**a, b**), both the 50-keV (**c, d**) and DL-based iodine-boosting reconstruction (**e, f**) demonstrated significantly increased contrast between the hepatic vessels (arrowheads) and enhanced main portal vein visualization (asterisk)

In the RPP group, iDose imaging displayed significantly lower image contrast (2.39 ± 0.40) than 50-keV imaging (3.60 ± 0.65) and DL-based iodine-boosting imaging (3.75 ± 0.60) during the arterial phase, with similar results in the portal venous phase (iDose, 2.41 ± 0.47; 50-keV, 4.01 ± 0.49; DL-based iodine-boosting, 3.86 ± 0.42) (*P* < 0.001 for all, Table E2) (Fig. 5). The overall image quality was also significantly lower in iDose images compared to the other imaging methods (*P* < 0.001, Table E2). No significant differences were observed between the 50-keV images and the DL-iodine-boosting images in terms of image noise and image contrast during both arterial and portal venous phases (*P* > 0.017). Image textures were significantly altered in the DL-iodine-boosting images compared to the 50-keV images. However, in quantitative analyses, the SNR of the aorta, the SNR of the portal vein, and the CNR of the liver were highest in the DL-iodine-boosting images, followed by the 50-keV images and then the iDose images (Table E2).

### Post-contrast renal function follow-up

No participant reported a decrease in urine output within 48 h after CECT. Only 11 participants (6 in the standard group and 5 in the RPP group) underwent a follow-up serum creatinine test within 1 month (median, 13 days; range, 4–25 days). The mean difference in serum creatinine level from before to after the CT examinations was 0.07 ± 0.09 mg/dL (range, 0.0–0.23) in the standard group and 0.006 ± 0.009 mg/dL (0.0–0.02) in the RPP group (*P* = 0.370). The relative difference in post-CT serum creatinine levels from baseline was 4.39% ± 6.24% (range − 2.16–10.9%) in the standard group and 0.60% ± 0.83% (range, − 0.42–1.63%) in the RPP group (*P* = 0.370).

## Discussion

Our study demonstrated that the diminished image contrast resulting from a reduction in iodinated CM could be restored through 50-keV reconstruction or DL-based iodine-boosting techniques. These alternative image reconstruction methods were particularly effective in participants with impaired renal function. Images from the RPP group with 50-keV reconstruction or the DL-based iodine-boosting techniques exhibited higher image contrast scores in both arterial and portal venous phases compared to the standard group, despite a 43% reduction in CM dose. Furthermore, both the 50-keV and DL-based iodine-boosting techniques in the RPP group provided superior overall image quality compared to the standard group for both phases. These findings suggest that the RPP protocol can potentially improve image quality through advanced reconstruction techniques, such as 50-keV imaging and DL-iodine-boosting, even with a smaller CM dose. This is particularly important for patients with kidney diseases or other conditions for which the administration of CM is a concern. Ensuring patient safety is especially crucial for these individuals, as they often require repeated scans as part of their oncological care. Considering that our study population consisted of patients at risk of developing HCC who had impaired renal function, minimizing the contrast dose administered during CT examinations is crucial. We firmly believe that our results support the effectiveness of our RPP protocol, which combines a reduced CM dose and less conventional image reconstruction techniques, for enhancing patient safety while still maintaining adequate image quality for diagnostic purposes.

In our study, we compared the image quality of the standard group with hybrid iterative reconstruction and the RPP group with DL-based iodine-boosting reconstruction, as well as low monoenergetic images (50 keV). Notably, the subjective image quality was significantly better in the DL-based iodine-boosting reconstruction images, despite the reduction in CM dose, which is consistent with a recent study [19]. In addition, we intentionally used low iodine concentration CM to ensure adequate arterial phase given the significant reduction of contrast volume in RPP group. To date, most studies aimed at reducing CM dose have utilized low kV to boost iodine contrast, which offers the additional benefit of a reduced radiation dose [12, 24]. However, low-kV imaging has the disadvantage of potentially increasing the radiation dose in larger patients, and its use is limited by increased noise and reduced image quality. Therefore, low-kV imaging is primarily used for either CT angiography or low-dose chest CT, in which acceptable noise levels are relatively high due to the inherently high CNR between the vessels and background or the focal lesions and air. The main purpose of low-kV imaging is to reduce radiation dose rather than the CM dose.

Recent technical developments have demonstrated another approach to enhance the contrast resolution in CECT. In these studies, low monoenergetic images (< 70 keV) could boost iodine contrast and provide higher SNR and CNR [13, 14]. However, the use of low monoenergetic images remains limited because the main workhorse in clinical practice is still the single-energy scanner. Additionally, low monoenergetic images (< 70 keV) may not be adequately available on older dual-energy CT scanners due to insufficient noise suppression, which arises from the unavailability of noise reduction techniques such as the spatial frequency separation algorithm or anti-correlated noise reduction [16–18]. An important point in this context is that the DL-based iodine-boosting reconstruction method used in this study is vendor-neutral. It can increase image contrast regardless of the scanner type and reconstruction technique, thus offering the advantage of superior generalizability. Therefore, our study results demonstrate the potential use of this low-CM dose protocol in general, without the requirement of specific conditions such as certain vendors or scanner specifications.

Although image quality improved, we did not observe a significant difference in lesion conspicuity and lesion detection rates between the standard CT images and 50-keV or DL-based iodine-boosting reconstruction images in the RPP group. Both 50-keV and DL-based iodine-boosting reconstruction in the RPP group tended to display higher lesion conspicuity than the standard group in both arterial and portal venous phases, but this finding was not statistically significant. This may have been due to the relatively small number of FLLs in each group and the associated low statistical power. Since we could not predict the occurrence of HCC before CT examination, the discrepancy of the FLL size and number between the groups caused a challenge of simple comparison for diagnostic performance. Despite the statistical insignificance, we cautiously suggest that the tendency for similar to higher lesion conspicuity in the RPP group on size-stratified comparison implies that diagnostic performance might not be compromised by the use of a reduced CM dose.

Notably, in the intra-individual comparison between 50-keV and DL-based iodine-boosting reconstructed images, the DL-based iodine-boosting reconstructed images exhibited higher SNR and CNR in both the arterial and portal venous phases than the 50-keV images. However, the 50-keV images demonstrated better subjective image quality and higher lesion conspicuity than the DL-based iodine-boosting images. This may have been due to the alteration of image texture in DL-based iodine-boosting images. As discussed above, DL-based iodine-boosting was performed in two steps, and the initial denoising step may introduce additional texture alteration compared to low monoenergetic images. Further studies are needed to determine the effects of reduced CM and reconstruction methods on FLL detection.

Recently developed techniques, such as dual-energy CT and DL-based image reconstruction, have been directed towards improving not only the performance of CECT, but also examination-related safety. Our study results, which demonstrate restored image contrast and quality, suggest that a low CM dose protocol could be prioritized for patients at risk of developing AKI. The necessity of this low CM dose protocol may be debatable due to a recent paradigm shift regarding contrast-induced AKI. Recent studies have shown that the risk of CM for inducing AKI has been overstated by comorbidities and high-osmolar CM, which is no longer used. As a result, recent guidelines have relaxed the criteria for instances requiring prophylaxis for AKI. Even the American College of Radiology-National Kidney Foundation has stated that a reduced CM dose is not specifically required for those at risk of AKI due to concerns about suboptimal image quality, and a single diagnostic dose should be administered [4]. We agree with this statement because an unnecessarily high CM dose should be avoided in any patient, regardless of their renal function, and our study results can be easily applied to populations without renal dysfunction.

In addition to reducing the risk of contrast-associated or contrast-induced AKI, lowering the CM dose can potentially decrease the risk of extravasation and hypersensitivity reactions, which have been reported to be CM dose-related [25, 26]. Iodinated CM has been reported to increase the absorbed radiation dose [27, 28]. Therefore, using a low dose of CM is a crucial step in minimizing contrast-related side effects as well as medical radiation exposure. Furthermore, the only possible downside of reducing the iodinated CM dose is the concern regarding suboptimal image quality. With this in mind, we firmly believe that exploring the lower limit of iodinated CM reduction is essential. However, the cut-off value of a single diagnostic CM dose has not yet been clearly established and may vary across patients and CT protocols. Additionally, recent studies have focused on individualizing CT protocols—that is, it may be necessary to determine on a case-by-case basis whether reducing the radiation dose or avoiding the risk of contrast-associated nephropathy is more important [29]. We should be cautious not to exclude patients solely based on the eGFR, as the risk of contrast-associated AKI is estimated to be between 10% and 30% depending on the eGFR [4]. Comorbidity-related AKI risks are challenging to estimate, and the eGFR sometimes exhibits high variability [30].

Our study had several limitations. First, the small number of participants could affect the statistical power. Although the sample size was calculated for the primary endpoint, the low incidence of non-cystic FLLs might have weakened the statistical significance of comparisons between the standard and RPP groups in terms of lesion conspicuity and lesion detection. Furthermore, we had an imbalance of FLL size and number between the two groups. It is inevitable since we could not predict which patients would have HCC at the time of enrollment. Second, most participants had moderate renal dysfunction, as patients with significantly decreased eGFR (< 30 mL/min/1.73 m^2^) do not undergo liver CT, and alternative examinations such as contrast-enhanced ultrasound or non-contrast MRI are often considered for those patients according to our institutional policy. However, we believe that the patients with moderate renal dysfunction still belong to a concerning group since they often had multiple other risk factors, such as diabetes or nephrotoxic medication use. Third, we used only a dual-layer CT scanner from a single vendor, which may limit the generalizability of our findings to other types of scanners. Lastly, follow-up serum creatinine values were only available for a few patients, and we did not observe a significant difference in serum creatinine levels between the groups. The clinical impact of a reduced CM dose on contrast-induced nephropathy in patients with decreased renal function remains unknown and should be investigated in future studies.

## Conclusion

The reduction of iodinated CM, which leads to decreased image contrast and overall image quality, can be compensated for by using either low monoenergetic imaging or DL-based iodine-boosting reconstruction methods. A low iodine dose of 300 mgI/kg can be compensated for by low monoenergetic imaging or DL-based iodine-boosting techniques.

### Electronic supplementary material

Below is the link to the electronic supplementary material.

Supplementary Material 1

Supplementary Material 2

## Data Availability

The datasets used and/or analysed during the current study are available from the corresponding author on reasonable request.

## Abbreviations

AKI: Acute kidney injury
CECT: Contrast-enhanced computed tomography
CM: Contrast medium
CNR: Contrast-to-noise ratio
DL: Deep learning
eGFR: Estimated glomerular filtration rate
FLL: Focal liver lesion
HCC: Hepatocellular carcinoma
RPP: Renal protection protocol

## References

[1] Villanueva A (2019). Hepatocellular Carcinoma. N Engl J Med.

[2] Kim Y, Lim HK, Rhim H, Lee MW, Choi D, Lee WJ (2013). Ten-year outcomes of percutaneous radiofrequency ablation as first-line therapy of early hepatocellular carcinoma: analysis of prognostic factors. J Hepatol.

[3] Ichikawa T, Okada M, Kondo H, Sou H, Murakami T, Kanematsu M (2013). Recommended iodine dose for multiphasic contrast-enhanced mutidetector-row computed Tomography Imaging of Liver for assessing Hypervascular Hepatocellular Carcinoma: Multicenter prospective study in 77 General Hospitals in Japan. Acad Radiol.

[4] Davenport MS, Perazella MA, Yee J, Dillman JR, Fine D, McDonald RJ (2020). Use of Intravenous Iodinated contrast media in patients with kidney disease: Consensus statements from the American College of Radiology and the national kidney Foundation. Radiology.

[5] D’Amico G, Garcia-Tsao G, Pagliaro L (2006). Natural history and prognostic indicators of survival in cirrhosis: a systematic review of 118 studies. J Hepatol.

[6] du Cheyron D, Bouchet B, Parienti J-J, Ramakers M, Charbonneau P (2005). The attributable mortality of acute renal failure in critically ill patients with liver cirrhosis. Intensive Care Med.

[7] Heimbach JK, Kulik LM, Finn RS, Sirlin CB, Abecassis MM, Roberts LR (2018). AASLD guidelines for the treatment of hepatocellular carcinoma. Hepatol.

[8] Galle PR, Forner A, Llovet JM, Mazzaferro V, Piscaglia F, Raoul J-L (2018). EASL Clinical Practice Guidelines: management of hepatocellular carcinoma. J Hepatol.

[9] Hatzaras I, Bischof DA, Fahy B, Cosgrove D, Pawlik TM (2014). Treatment Options and Surveillance Strategies after Therapy for Hepatocellular Carcinoma. Ann Surg Oncol.

[10] Singal AG, Llovet JM, Yarchoan M, Mehta N, Heimbach JK, Dawson LA (2023). AASLD Practice Guidance on prevention, diagnosis, and treatment of hepatocellular carcinoma. [published online ahead of print, 2023 May 22]. Hepatol.

[11] Runge VM (2017). Critical questions regarding Gadolinium Deposition in the brain and body after injections of the Gadolinium-Based contrast agents, Safety, and clinical recommendations in consideration of the EMA’s Pharmacovigilance and Risk Assessment Committee recommendation for suspension of the marketing authorizations for 4 Linear Agents. Invest Radiol.

[12] Kayan M, Köroğlu M, Yeşildağ A, Ceylan E, Aktaş AR, Yasar S (2012). Carotid CT-angiography: low versus standard volume contrast media and low kV protocol for 128-slice MDCT. Eur J Radiol.

[13] Yoon JH, Chang W, Lee ES, Lee SM, Lee JM (2020). Double low-dose dual-energy liver CT in patients at high-risk of HCC: a prospective, randomized, single-center study. Invest Radiol.

[14] Nagayama Y, Nakaura T, Oda S, Utsunomiya D, Funama Y, Iyama Y (2018). Dual-layer DECT for multiphasic hepatic CT with 50% iodine load: a matched-pair comparison with a 120 kVp protocol. Eur Radiol.

[15] Oda S, Takaoka H, Katahira K, Honda K, Nakaura T, Nagayama Y (2019). Low contrast material dose coronary computed tomographic angiography using a dual-layer spectral detector system in patients at risk for contrast-induced nephropathy. Br J Radiol.

[16] Kalisz K, Rassouli N, Dhanantwari A, Jordan D, Rajiah P (2018). Noise characteristics of virtual monoenergetic images from a novel detector-based spectral CT scanner. Eur J Radiol.

[17] Li Z, Yu L, Trzasko JD, Lake DS, Blezek DJ, Fletcher JG (2014). Adaptive nonlocal means filtering based on local noise level for CT denoising. Med Phys.

[18] Grant KL, Flohr TG, Krauss B, Sedlmair M, Thomas C, Schmidt B (2014). Assessment of an Advanced Image-Based technique to calculate virtual Monoenergetic computed Tomographic images from a dual-energy examination to improve Contrast-To-Noise ratio in examinations using Iodinated contrast media. Invest Radiol.

[19] Kang H-J, Lee JM, Ahn C, Bae JS, Han S, Kim SW (2023). Low dose of contrast agent and low radiation liver computed tomography with deep-learning-based contrast boosting model in participants at high-risk for hepatocellular carcinoma: prospective, randomized, double-blind study. Eur Radiol.

[20] Levey AS, Stevens LA, Schmid CH, Zhang Y (Lucy), Castro AF, Feldman HI A New Equation to Estimate Glomerular Filtration Rate, et al. editors. Ann Intern Med. 2009;150:604–12. 10.7326/0003-4819-150-9-200905050-00006 PMC2763564 19414839

[21] Lee T, Lee JM, Yoon JH, Joo I, Bae JS, Yoo J (2022). Deep learning–based image reconstruction of 40-keV virtual monoenergetic images of dual-energy CT for the assessment of hypoenhancing hepatic metastasis. Eur Radiol.

[22] Bae JS, Lee JM, Kim SW, Park S, Han S, Yoon JH (2023). Low-contrast-dose liver CT using low monoenergetic images with deep learning–based denoising for assessing hepatocellular carcinoma: a randomized controlled noninferiority trial. Eur Radiol.

[23] Hausleiter J, Martinoff S, Hadamitzky M, Martuscelli E, Pschierer I, Feuchtner GM (2010). Image quality and Radiation exposure with a low tube voltage protocol for coronary CT angiography: results of the PROTECTION II trial. JACC: Cardiovasc Imaging.

[24] Araki K, Yoshizako T, Yoshida R, Tada K, Kitagaki H (2018). Low-voltage (80-kVp) abdominopelvic computed tomography allows 60% contrast dose reduction in patients at risk of contrast-induced nephropathy. Clin Imaging.

[25] Park HJ, Son JH, Kim T-B, Kang MK, Han K, Kim EH (2019). Relationship between lower dose and injection speed of Iodinated contrast material for CT and Acute hypersensitivity reactions: an observational study. Radiology.

[26] Liu H, Qiu H, Liu J, Wang L, Zhao L, Wang Y (2023). Stratified assessment and warning regimen for prevention of acute adverse reactions to iodinated contrast media: results of 150,343 cases in a tertiary hospital. Med Biol Eng Comput.

[27] Mazloumi M, Van Gompel G, Kersemans V, de Mey J, Buls N (2021). The presence of contrast agent increases organ radiation dose in contrast-enhanced CT. Eur Radiol.

[28] Perisinakis K, Tzedakis A, Spanakis K, Papadakis AE, Hatzidakis A, Damilakis J (2018). The effect of iodine uptake on radiation dose absorbed by patient tissues in contrast enhanced CT imaging: implications for CT dosimetry. Eur Radiol.

[29] Martens B, Jost G, Mihl C, Nijssen EC, Wildberger JE, Schmidt B (2022). Individualized scan protocols in Abdominal computed tomography: Radiation Versus contrast Media Dose optimization. Invest Radiol.

[30] Björk J, Nyman U, Courbebaisse M, Couzi L, Dalton RN, Dubourg L (2020). Prospects for improved glomerular filtration rate estimation based on creatinine—results from a transnational multicentre study. Clin Kidney J.

