# Supplementary material for: Renal protection CT protocol using low-dose and low-concentration iodine contrast medium in at-risk patients of HCC and with chronic kidney disease: a randomized controlled non-inferiority trial
Source: Cancer Imaging. 2023 Oct 19;23:100. doi: 10.1186/s40644-023-00616-0 (PMC10588122; doi:10.1186/s40644-023-00616-0)
Supplement: Supplementary file 1 — Supplementary Material 1 [file 40644_2023_616_MOESM1_ESM.docx]

**SUPPLEMENT**

**Reference standard for focal liver lesions**

*Hepatocellular carcinoma—* For hepatocellular carcinoma (HCC), diagnosis was made using a composite algorithm. The pathologic diagnosis was used regardless of imaging features. Lesions were diagnosed as HCCs on imaging basis in the following cases: a) Liver Imaging Reporting and Data System (LI-RADS) scores 4-5 (LR-4 or -5) with tumor staining on angiography for transarterial chemoembolization (TACE), definite nodule on ultrasound (US) and LR-4 or -5 on contrast-enhanced US; b) tumor-in-vein (TIV) with arterial phase hyperenhancement (APHE) and portal washout. Observations with LR-M features were regarded as HCC if alpha-feto protein or PIVKA elevation developed together. For HCCs (n = 40), diagnoses were made by tumor staining on angiography and subsequent lipiodol uptake in (30%, 12/40), LR-4 or LR-5 nodules on follow-up computed tomography (CT) in (47.5%, 19/40) or follow-up magnetic resonance imaging (MRI) (22.5%, 9/40).

*Cirrhotic nodule—* Cirrhotic nodules were diagnosed for observations showing following imaging features and stable on follow-up images: a) hepatobiliary phase defects on MRI without APHE; b) hepatobiliary hyperintense nodules on MRI without APHE; c) persistent low attenuation on portal and delayed phase of CT without APHE. For cirrhotic nodules (n = 19), aforementioned imaging features were shown and stable for follow-up CT (n = 4) or MRI (n = 15).

*Hemangiomas—* Hemangiomas were clinically diagnosed based on its characteristic features including bright intensity on T2-weighted imaging on magnetic resonance imaging (MRI) and the peripheral nodular enhancement pattern on CECT or dynamic MRI as well as no significant interval change during follow-up. One hemangioma showed typical imaging features on MRI.

*Post-inflammatory granulomas*— Post-inflammatory granuloma (n = 1) was diagnosed from previous serial exams showing regressed liver abscess after antibiotic treatment.

*Vascular malformation*— Vascular malformation with portal vein branch aneurysmal dilatation was diagnosed based on connection between hepatic vein and portal vein with engorged vessels and early opacification of hepatic vein. The lesion showed stability during follow-up (n = 1).

**Focal liver lesions in each group**

In standard group, 47 non-cystic focal liver lesions (FLLs) were found in 11 participants, including 61.7% hepatocellular carcinoma (29/47, 17.7± 18.3 mm, range 5–89 mm), 31.9% cirrhotic nodule (15/47, 11.3 ± 3.3 mm, 8–20 mm), 2.1% hemangioma (1/47, 5 mm), 2.1% post-inflammatory granuloma (1/47, 12 mm) and 2.1% vascular malformation (1/47, 13 mm).

In renal protection protocol group, 15 non-cystic FLLs were observed in 6 participants, including 73.3% HCC (11/15, 29 ± 22 mm, range 9–76 mm) and 26.7% cirrhotic nodule (4/15, 17.5 ± 5.9 mm, range 12–24 mm).

**Table E1. Scale of Qualitative Image Analysis**

| **Items** | **Scale** | **Descriptions** |
| --- | --- | --- |
| Image noise | 1–5 | Score 1, undiagnostic image  Score 2, significant image noise affecting diagnostic confidence  Score 3, diagnostically acceptable but noticeable image quality decrease  Score 4, mild image noise and no or mild image quality decrease  Score 5, no definite image noise, similar to model-based iterative reconstruction |
| Image contrast | 1–5 | Score 1, substantial lack of contrast similar to non-contrast CT or nephrogenic phase  Score 2, poor contrast  Score 3, average contrast  Score 4, good contrast  Score 5, very strong contrast of the images |
| Image texture | 1–5 | Score 1, undiagnostic image  Score 2, significant artificial sensation including blurring, alteration of image texture affecting diagnostic confidence  Score 3, diagnostically acceptable but noticeable artificial sensation by image blurring and/or alteration of image texture  Score 4, mild artificial sensation by alteration of image texture, and no or mild image quality decrease  Score 5, no definite artificial sensation by image texture alteration |
| Overall image quality | 1–5 | Score 1, undiagnostic image  Score 2, poorer than average but does not require re-examination  Score 3, average  Score 4, better than average  Score 5, excellent |

**Table E2. Comparisons of three image reconstruction algorithms in Renal Protection Protocol group**

|  | **iDose reconstruction (A)** | **50 keV reconstruction (B)** | **DL-iodine boosting (C)** | ***P*-value** | | |
| --- | --- | --- | --- | --- | --- | --- |
|  |  |  |  | **A vs. B** | **A vs. C** | **B vs. C** |
| **Arterial phase** | | | | | | |
| Qualitative Image noise | 3.32 ± 0.42 (2.00, 4.25) | 3.93 ± 0.45(2.50, 4.75) | 3.89 ± 0.42(2.50, 4.75) | < 0.001 | < 0.001 | > 0.999 |
| Quantitative Image noise | 10.47 ± 1.36 (7.80, 14.11) | 8.66 ± 1.28 (6.20, 11.42) | 7.74 ± 0.95 (6.26, 10.01) | < 0.001 | < 0.001 | 0.019 |
| Image contrast | 2.39 ± 0.40 (1.75, 3.75) | 3.60 ± 0.65 (1.75, 4.50) | 3.75 ± 0.60 (1.75, 4.50) | < 0.001 | < 0.001 | > 0.999 |
| SNR of aorta | 23.42 ± 5.79 (11.75, 36.33) | 57.09 ± 14.29 (24.81, 78.55) | 67.45 ± 17.66 (26.34, 100.75) | < 0.001 | < 0.001 | 0.019 |
| Image texture | 4.30 ± 0.35 (3.50, 4.75) | 4.06 ± 0.32(3.50, 4.50) | 3.56 ± 0.53(3.50, 4.50) | 0.107 | < 0.001 | < 0.001 |
| Overall image quality | 2.68 ± 0.40 (2.00, 3.50) | 3.88 ± 0.58 (2.25, 4.75) | 3.57 ±0.56 (2.25, 4.50) | < 0.001 | < 0.001 | 0.101 |
| **Portal venous phase** | | | | | | |
| Qualitative Image noise | 3.42 ± 0.29 (2.75, 3.75) | 4.12 ±0.29 (3.50, 4.75) | 3.98 ± 0.27(3.25, 4.50) | < 0.001 | < 0.001 | 0.220 |
| Quantitative Image noise | 10.61 ± 1.43 (8.26, 14.45) | 9.13 ± 1.76 (6.65, 13.40) | 8.04 ± 1.05 (6.61, 11.04) | 0.001 | < 0.001 | 0.022 |
| Image contrast | 2.41 ± 0.47 (1.75, 4.00) | 4.01 ± 0.49 (2.25, 4.75) | 3.86 ± 0.42 (3.25, 4.75) | < 0.001 | < 0.001 | 0.731 |
| SNR of portal vein | 11.65 ± 2.35 (7.67, 16.87) | 24.71 ± 5.70 (15.11, 36.54) | 29.29 ± 6.30 (19.15, 43.83) | < 0.001 | < 0.001 | 0.004 |
| SNR of liver | 8.74 ± 1.63 (5.61, 12.62) | 14.61 ± 3.39 (6.60, 21.33) | 14.61 ± 3.39 (6.60, 21.33) | < 0.001 | < 0.001 | 0.023 |
| CNR of liver | 2.91 ± 1.17 (4.36, 4.88) | 10.11 ± 3.01 (5.56, 15.21) | 12.34 ± 3.19 (5.29, 19.45) | < 0.001 | < 0.001 | 0.007 |
| Image texture | 4.37 ± 0.31 (3.75, 4.75) | 4.09 ± 0.23(3.75, 4.50) | 3.50 ± 0.42 (3.00, 4.50) | 0.009 | < 0.001 | < 0.001 |
| Overall image quality | 2.69 ± 0.39 (2.00, 3.50) | 4.16 ±0.35 (3.25, 4.75) | 3.63 ± 0.44 (3.00, 4.50) | < 0.001 | < 0.001 | < 0.001 |

Note: CNR = contrast-noise-ratio, DL = deep learning, SNR = signal-to-noise ratio. Global *P*-values were < 0.001 for all variables. *P*-value < 0.017 was considered to indicate statistically significant difference in pairwise comparisons.
